# Genomic organization, sequence divergence, and recombination of feline immunodeficiency virus from lions in the wild

**DOI:** 10.1186/1471-2164-9-66

**Published:** 2008-02-05

**Authors:** Jill Pecon-Slattery, Carrie L McCracken, Jennifer L Troyer, Sue VandeWoude, Melody Roelke, Kerry Sondgeroth, Christiaan Winterbach, Hanlie Winterbach, Stephen J O'Brien

**Affiliations:** 1 Laboratory of Genomic Diversity, National Cancer Institute-Frederick, Frederick MD 21702 USA; 2 Laboratory of Genomic Diversity, Basic Research Program-SAIC Frederick – National Cancer Institute, Frederick, MD 21702 USA; 3 Department of Microbiology, Immunology and Pathology, Colorado State University, Fort Collins CO 80532 USA; 4 Department of Veterinary Microbiology and Pathology, Washington State University, Pullman WA 99164 USA; 5 Tau Consultants, Private Bag 83, Maun, Botswana

## Abstract

**Background:**

Feline immunodeficiency virus (FIV) naturally infects multiple species of cat and is related to human immunodeficiency virus in humans. FIV infection causes AIDS-like disease and mortality in the domestic cat (*Felis catus*) and serves as a natural model for HIV infection in humans. In African lions (*Panthera leo*) and other exotic felid species, disease etiology introduced by FIV infection are less clear, but recent studies indicate that FIV causes moderate to severe CD4 depletion.

**Results:**

In this study, comparative genomic methods are used to evaluate the full proviral genome of two geographically distinct FIV subtypes isolated from free-ranging lions. Genome organization of FIV_*Ple *_subtype B (9891 bp) from lions in the Serengeti National Park in Tanzania and FIV_*Ple *_subtype E (9899 bp) isolated from lions in the Okavango Delta in Botswana, both resemble FIV genome sequence from puma, Pallas cat and domestic cat across 5' LTR, *gag, pol, vif, orfA, env, rev *and 3'LTR regions. Comparative analyses of available full-length FIV consisting of subtypes A, B and C from FIV_*Fca*_, Pallas cat FIV_*Oma *_and two puma FIV_*Pco *_subtypes A and B recapitulate the species-specific monophyly of FIV marked by high levels of genetic diversity both within and between species. Across all FIV_*Ple *_gene regions except *env*, lion subtypes B and E are monophyletic, and marginally more similar to Pallas cat FIV_*Oma *_than to other FIV. Sequence analyses indicate the SU and TM regions of *env *vary substantially between subtypes, with FIV_*Ple *_subtype E more related to domestic cat FIV_*Fca *_than to FIV_*Ple*_ subtype B and FIV_*Oma *_likely reflecting recombination between strains in the wild.

**Conclusion:**

This study demonstrates the necessity of whole-genome analysis to complement population/gene-based studies, which are of limited utility in uncovering complex events such as recombination that may lead to functional differences in virulence and pathogenicity. These full-length lion lentiviruses are integral to the advancement of comparative genomics of human pathogens, as well as emerging disease in wild populations of endangered species.

## Background

Feline immunodefiency viruses (FIV) naturally infect cat species in the wild and are related to other lentiviruses known to infect primates (human and simian immunodeficiency viruses, HIV and SIV), sheep and goats (caprine arthritis encephalitis virus -CAEV), horse (equine infectious anemia virus-EIAV), and cattle (bovine immunodeficiency virus-BIV). FIV is endemic in Felidae species [1-12], many of which are considered endangered or threatened with extinction [13]. A recent comprehensive survey of serum and lymphocyte specimens from 3055 individuals affirm that at least 11 free-ranging and, if captive animals are included, as many as 31 species of cat are infected with FIV [4]. Phylogenetic analyses of the *pol-RT *region sequenced from six of these felid species, plus spotted hyaena, *Crocuta crocuta*, affirm the high level of species-specificity worldwide [4,11,14-17]. Each species specific FIV forms a distinct monophyletic lineage, separated by substantial genetic divergence that suggests virus-host adaptation and rare episodes of interspecies transmission in the wild [4,18].

The effects of FIV infection and disease are well described in domestic cat (*Felis catus*) but less so in exotic felids. FIV_*Fca*_ infection in domestic cat is analogous to HIV infection of humans causing early flu-like symptoms, followed by severe weight loss, chronic wasting disease, and increased susceptibility to rare cancers and opportunistic disease, neurologic disease and death [19,20]. Captive and wild populations of two species, the African lion (*Panthera leo*) infected with FIV_*Ple *_and the puma (*Puma concolor*), infected with FIV_*Pco *_exhibit less severe disease associations. However, infected lions show a dramatic decline in CD4+ subsets, a reduction of the CD4+/CD8+ ratio, reduction of CD8+β^high ^cells, and expansion of the CD8+β^low ^subset relative to uninfected lions [21-23]. Further, FIV_*Pco *_infected puma display a more generalized response of lymphopenia expressed as a significant decline in total lymphocytes, CD5+ T-cells, and CD5- lymphocytes as well as a significant reduction in CD4+ T-cells [23]. Like lions, seropositive pumas have a significant decline in CD8+β^high ^cells but differ by not showing compensatory expansion of CD8+β^low ^cells relative to controls [23]. The results observed with FIV-infected lion and puma parallels human (HIV) and Asian monkey (SIV) CD4+ diminution, and suggests there may be an immunological cost of FIV infection in these two species of large cats.

Identification of genetic correlates of FIV virulence, infectivity, and pathogenicity in different cat species is limited due to a paucity of complete genome sequence. Only subtypes A, B and C from domestic cat FIV_*Fca *_[24-26], subtypes A and B from puma FIV_*Pco*_[14,27] and a single strain (FIV_*Oma*_) from Pallas cat (*Otocolobus manul*) [16] have been sequenced in entirety. Here we present full-length provirus sequenced from FIV_*Ple *_subtype B isolated from lions in the Serengeti National Park in Tanzania and FIV_*Ple *_subtype E from lions dwelling in the Okavango Delta in Botswana. These two FIV_*Ple *_subtypes exhibit a range in sequence divergence throughout the genome, share motifs unique to this lion-specific lentivirus, yet also exhibit unusual and significant differences in the *env *gene.

## Results and Discussion

### Genomic Organization and Sequence Divergence of FIV_*Ple *_Subtypes

FIV_*Ple *_subtypes B (accession number EU117991) and E (accession number EU117992) share a similar genome organization with other FIV which consists of LTR, *gag*, *pol*, *vif*, *orfA*, *env*, and additional small ORFs that may represent accessory genes including *rev *(Table 1). The total proviral genome size was conserved between FIV_*Ple *_subtype B (9899 bp) and subtype E (9891 bp) (Table 1). FIV_*Ple *_*gag *encodes three putative structural proteins of matrix, capsid and nucleocapsid. *Pol *is conserved and encodes key viral enzymes of protease, reverse transcriptase, RNAase, dUTPase and integrase. FIV_*Ple *_*vif*, an accessory protein essential for viral replication, resembles that of FIV_*Fca*_. *OrfA *in FIV_*Ple *_is similar to FIV_*Fca *_and likely corresponds to HIV *tat*, which targets transcription factors in the LTR. FIV_*Ple *_*env *encodes the putative leader, surface (SU), and transmembrane (TM) regions of the envelope glycoprotein, essential components for viral binding to and entry into the host cell. FIV_*Ple *_*rev *is similar to HIV/FIV *rev*, and is thought to be critical in viral replication. FIV_*Ple *_*rev *appears to be encoded by splicing two exons: the first in the leader region of *env*, the second located near the 3' region adjacent to *env *(Table 1).

**Table 1:** Gene size and location within FIV_*Ple *_Subtypes B and E compared with previously published FIV_*Fca*_, FIV_*Oma *_and FIV_*Pco*_.

	5'LTR	5'UTR	Gag	Pol	Vif	OrfA	Env	PPT	3'LTR
FIV_*Ple *_Subtype B (Serengeti)									
Gene position	1–398	399–704	705–2213	2018–5464	5461–6171	6288–6542	6601–9213	9484–9498	9501–9899
Gene length (bp)	398	306	1509	3447	711	255	2613	15	398
Translated Protein Size (# aa)			503	1149	237	85	871		
FIV_*Ple *_Subtype E (Botswana)									
Gene Position	1–397	398–702	703–2199	2004–5450	5447–6211	6198–6452	6532–9222	9478–9492	9495–9891
Gene length (bp)	397	306	1497	3447	765	255	2691	15	397
Translated Protein Size (# aa)			498	1149	255	85	897		
FIV_*Fca *_Petaluma (Subtype A)									
Gene Position	1–355	356–627	628–1980	1868–5243	5236–5991	5992–6228	6266–8836	9098–9117	9120–9474
Gene length (bp)	355	272	1353	3375	756	237	2571	19	
Translated Protein Size (# aa)			451	1125	252	79	857		
FIV_*Fca *_USIL (Subtype B)									
Gene position	1–361	362–633	634–1983	1875–5248	5239–5994	5995–6231	6269–8830	9092–9110	9102–9462
Gene length (bp)	361	272	1350	3374	756	237	2562	17	361
Translated Protein Size (# aa)			451	1124	252	79	854		
FIV_*Fca *_Subtype C									
Gene Position	1–354	355–632	633–1985	1874–5248	5241–5996	5997–6233	6271–8835	9092–9100	9113–9466
Gene length (bp)	354	278	1353	3375	756	237	2565	19	354
Translated Protein Size (# aa)			451	1125	252	79	855		
FIV_*Oma*_									
Gene Position	1–376	377–684	685–2181	1980–5432	5429–6187	6188–6448	6512–9103	9360–9375	9378–9751
Gene length (bp)	376	308	1497	3453	759	261	2592	16	374
Translated Protein Size (# aa)			499	1161	253	87	864		
FIV_*Pco *_PLV-14 Subtype A (Florida)									
Gene Position	1–311	312–615	616–2055	2199–5459	5419–6249	5759–5938	6250–8772	8771–8787	8790–9100
Gene length (bp)	311	304	1440	3261	831	180	2523	17	311
Translated Protein Size (# aa)			480	1087	277	59	841		
FIV_*Pco *_PLV-1695 Subtype B (British Columbia)									
Gene Position	1–306	307–638	639–2024	1886–5323	5298–6008	5972–6310	6283–8715	8772–8784	8787–9092
Gene length (bp)	306	332	1386	3438	711	339	2433	13	305
Translated Protein Size (# aa)			462	1146	237	113	811		

**Figure 1:**
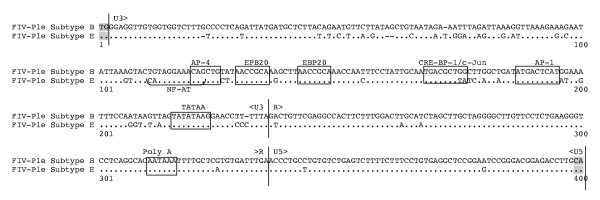
Alignment of FIV_*Ple *_subtype B and E LTR showing the U3, R and U5 regions. Grey shadow indicates inverted repeat, boxed regions indicate putative transcription elements common in FIV.

**Figure 2:**
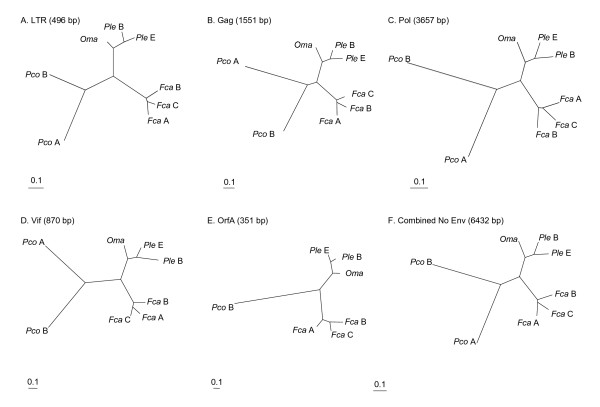
Phylogenetic reconstruction based on nucleotide sequence of LTR and coding genes from full-length FIV nucleotide sequences excluding *env*. (A-E) Shown are the maximum likelihood trees (ML) which are identical to tree topologies using maximum parisimony (MP) and minimum evolution (ME) for each gene region. See methods and Additional file 3 for specific parameters as implemented in PAUP ver 4.10b. (E) *OrfA *phylogeny does not include FIV_Pco _subtype A due to lack of sufficient homology for proper gene identification. (F) Phylogenetic tree of concatenated combined data of coding genes *gag*, *pol vif*, and *orfA*. All nodes supported by 100% bootstrap proportions in ME, MP and ML analyses except for relative positions of FIV_*Fca *_subtypes which were supported by bootstraps >50% but less than 100% within the FIV_*Fca *_clade.

The LTR of FIV_*Ple *_contains transcription and regulatory elements common to other FIV. These include the direct 2 bp repeat (IR) defining the 5' and 3' termini of LTR, AP-4, Aml-1 (EPB20), AP-1, TATA box, Poly A, and the cap transcription initiation site (Figure 1). *FIV*_*Ple *_subtypes have additional transcription factors characteristic of FIV, but placed in alternate locations within the LTR U3 including NF-AT and CREBP-1/c-Jun. These and other motifs were determined by homology search with a threshold value of 85% with the Motif Search database [28] [see Additional file 1]. Overall, lion LTRs are not identical between subtypes B and E, differing by 15% in nucleotide substitutions, comparable to that observed between FIV_*Fca *_subtypes A, B and C (Figure 1, Figure 2A).

Deep genetic divergence between FIV strains from different cat species made alignments problematic. For coding regions, we first translated each gene into amino acid residues, which are less divergent as changes occur at a lower rate of substitution, to serve as a "scaffold" for alignment of nucleotides using the program RevTrans [29]. Our results indicate that *pol *(3657 bp) is the most conserved gene across FIV, albeit exhibiting substantial average pair-wise genetic distances of 60% and 54% for nucleotide and amino acid data, respectively (Table 2). *Gag *sequences (1551 bp) differed by an average pairwise genetic distance of 65.8% for nucleotides, a 53.2% amino acids (Table 2). However, *vif *(870 bp)*, orfA *(351 bp), and *env *(2958 bp) were highly divergent. For these genes, sufficient homology existed to both identify the gene, and to create a multiple sequence alignment across all FIV yet, phylogenetic models for patterns of substitution at variable sites were saturated resulting in an average genetic distance of 100% for both nucleotide and amino acid data (Table 2). Such differences in rates of evolution between viral genes corroborate previous findings describing functional constraints for *gag *and *pol *[7,8,17], while also demonstrating that *vif, orfA*, and *env *rapidly evolve in each host species.

**Table 2:** Estimates of genetic divergence of FIV genes.

		FIV GENE				
		
		Gag	Pol	Vif	OrfA^1^	Env
		
Genetic distance						
		Nucleotide % Genetic Distance (GTR)				
Average Pairwise (N = 8)		65.8	60.3	100*	100*	100*
Selected comparisons						
	FIV_*Ple *_B vs FIV_*Ple *_E	20.3	20.4	33.1	27.9	100*
	FIV_*Ple *_B vs FIV_*Oma*_	28.8	29.3	44.3	55.8	42.7
	FIV_*Ple *_B vs FIV_*Fca *_C	55.6	53.5	100*	100*	100*
	FIV_*Ple *_E vs FIV_*Oma*_	32.9	28.1	36.7	61.3	100*
	FIV_*Ple *_E vs FIV_*Fca *_C	61.4	51.4	79.3	100*	64.4

		Amino Acid % Genetic Distance (Pam-Dayhoff^2^)				

Average Pairwise (N = 8)		53.2	44.1	100*	100*	100*
	FIV_*Ple *_B vs FIV_*Ple *_E	9.4	10.5	36.2	24.4	100*
	FIV_*Ple *_B vs FIV_*Oma*_	24.8	20.1	59.1	58.4	42.8
	FIV_*Ple *_B vs FIV_*Fca *_C	47.6	38.2	100*	100*	100*
	FIV_*Ple *_E vs FIV_*Oma*_	25.3	19.9	42.1	68.3	100*
	FIV_*Ple *_E vs FIV_*Fca *_C	46.2	37.9	91.9	100*	79.7

* 100% genetic distance means sufficient homology present to create alignment, but no meaningful phylogenetic associations are detected.

1 FIVPco A not included as no homologous OrfA identified.

2 See Methods

### Phylogenetic Analyses of FIV_*Ple *_Subtypes

The evolution of FIV_*Ple *_subtypes is defined by separate phylogenetic analyses of each viral gene as well as combined data of concatenated sequences representing the entire coding region of FIV. LTR, *gag*, *pol*, *vif *and *orfA *affirm the species-specificity of FIV both in individual gene analyses (Figure 2A–E) and in the combined concatenated data phylogeny excluding *env *(Figure 2F). The three subtypes of FIV_*Fca *_from the domestic cat exhibit the least amount of genetic divergence within each viral gene phylogeny. Sharing a monophyletic lineage with distantly related FIV_*Oma*_, the FIV_*Ple *_subtypes B and E have intermediate levels of genetic distance with each viral gene examined. Subtypes A and B of FIV_*Pco *_are the most divergent and have substantial differences across the viral genome. Thus, the hierarchical pattern of genetic divergence among full-length genomic analyses of FIV_*Fca*_, FIV_*Ple *_and FIV_*Pco *_recapitulates earlier evolutionary studies based on portions of *pol-RT *and *gag *[4,7,8,10-12,17,30,31].

The relative differences in genetic diversity among FIV strains may be correlated with the amount of time since the virus entered modern felids and therefore, can be interpreted in the context of the evolutionary and phylogeographic history of each host species. The domestic cat evolved as a unique felid lineage only around 10,000 year ago [32] from subspecies of wildcat *Felis silvestris *inhabiting Near East Asia [33]. Preliminary results from limited seroprevalence studies, indicate that FIV appears to be absent from nearly all of the close relatives of domestic cat [(genus *Felis *after [34]] except for French European wildcat *F. silvestris *[4,35]. Thus, the pattern of FIV_*Fca*_ divergence may represent recent emergence combined with rapid viral diversification within the domestic cat world-wide. In contrast, the puma is one of the oldest species within Felidae, sharing an evolutionary lineage with the African cheetah (*Acinonyx jubatus*) and the New World jaguarundi (*Puma yagouaroundi*) and arose approximately 4.5 MYA [34]. The extreme divergence between subtypes A and B within the FIV_*Pco *_lineage suggests an ancient origin of FIV infection of puma, a result consistent with the published *pol-RT *phylogeny marked by high levels of intra-subtype divergence of FIV_*Pco *_subtypes from throughout the host species range [4,8,11]. Lastly, the African lion species arose approximately 2 MYA and spread throughout Africa, Asia and the Americas [34]. However, due to episodes of population reduction followed by expansion from East Africa and recolonization, genomic diversity in modern lion populations coalesces to approximately 325,000 years ago and is confined the African continent [36]. FIV_*Oma *_is found in wild populations of the Eurasian Pallas cat [4], a species that arose during the late Pleistocene [34]. The monophyletic lineage of Pallas cat FIV_*Oma *_and African lion FIV_*Ple *_observed here suggest more ancient inter-species transmission as the last time lions and Pallas cats were in geographic contact was during the Pleistocene when lion ranges spread throughout Asia, providing a possible opportunity for FIV transmission between these species [37].

### Discordant *env *phylogeny between *FIV*_*Ple *_subtypes reveals ancestral FIV recombination events in the wild

**Figure 3:**
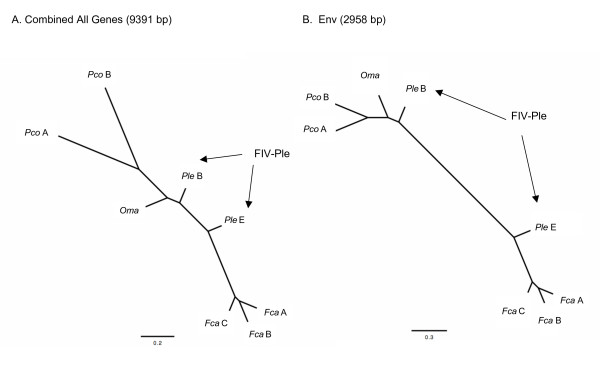
Phylogenetic reconstruction based on nucleotide sequence of fulllength proviral FIV including *env *and separate analysis of *env*. A. Phylogenetic tree of concatenated combined data of coding genes *gag*, *pol vif*, *orfA *and *env*. B. Phylogenetic tree of *env *sequences only. Shown is the maximum likelihood tree (ML) identical to tree topology using maximum parisimony (MP) and minimum evolution (ME) for each gene region. See methods and Additional file 3 for specific parameters as implemented in PAUP ver 4.10b. All nodes supported by 100% bootstrap proportions in ME, MP and ML analyses except for relative positions of FIV_*Fca *_subtypes which were supported by bootstraps >50% but less than 100% within the FIV_*Fca *_clade.

The patterns of phylogenetic divergence between FIV strains from different cat species are concordant between all viral gene regions with one notable exception, the *env *gene. Phylogenetic analyses of the entire concatenated coding region (9391 bp) and separate analysis of the *env *gene (2958 bp) show the two *FIV*_*Ple *_subtypes are no longer monophyletic (Figure 3A and 3B). A closer examination of the *env *gene shows only two shared regions of homology between FIV_*Ple *_subtypes. The first spans the sites 1–519 of *env*, containing exon 1 of *rev *(Table 1), within the leader region exhibiting 80% nucleotide and 68% amino acid homology between FIV_*Ple *_subtypes. The second region occurs at the terminal 3' region of *env *(sites 2506–2958) with 87% and 71% genetic identity for nucleotides and amino acid, respectively. Based on comparison with FIV_*Fca*_, this region of FIV_*Ple *_may be the *rev *responsive element (RRE), which is critical for targeting *rev *to the nucleolus of the cell [38]. As *rev *is conserved between lion subtypes, it is likely that RRE must remain conserved as well.

**Figure 4:**
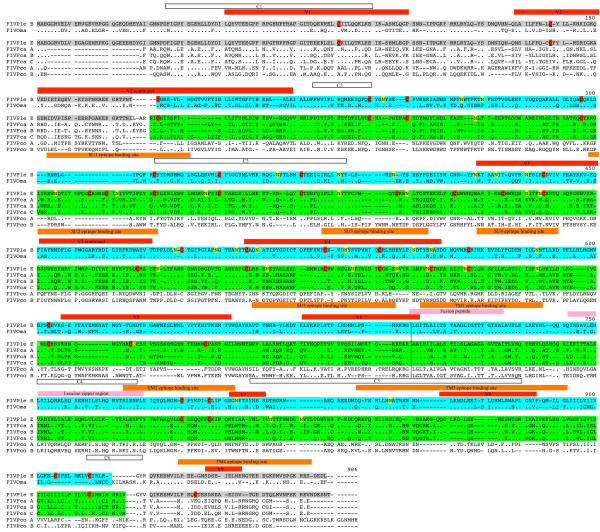
Multiple sequence alignment of amino acids of *env *from FIV_*Ple *_subtypes B and E compared with FIV_*Fca *_subtypes A, B and C, FIV_*Pco *_subtypes A and B, and FIV_*Oma*_. Significant structures within the *env *gene inferred from Smirnova et al. 2005 are indicated in colored boxes. Putative regions include: conserved amino acids (white box), variable regions V1-V9 (red box); epitope binding sites (orange box); conserved cysteine (red highlight); N glycosylation sites (yellow highlight). Homologous region shared between FIV_*Ple *_subtypes B and E are highlighted in grey. Amino acid sites 1–176 contain the first exon of *rev *(see Table 1) in lion FIV_*Ple *_subtypes B and E. The portion of *env *proposed to be a result of recombination in FIV_*Ple *_subtype E is highlighted in green. The corresponding region of *env *thought to represent FIV_*Ple *_without recombination, as it is more homologous to FIV_*Oma*_, is highlighted in blue. Amino acids sites 931–978 (grey) likely contain the RRE element shared by lion FIV_*Ple *_subtypes B and E.

By contrast, the SU and TM regions of *env *differ substantially between FIV_*Ple *_subtypes (Figure 4). A contiguous region of *env*, from amino acid sites 181 through 931 (green in Figure 4), shows that FIV_*Ple *_subtype E is more similar to FIV_*Fca *_than to *FIV*_*Ple *_subtype B. Further, *env *of FIV_*Ple *_subtype B, concordant with results from other gene trees (Figure 2A–E), shares more homology with FIV_*Oma *_(blue in Figure 4). Moreover, the lack of monophyly between FIV_*Ple *_subtype B and FIV_*Oma *_(Figure 3) is a consequence of the recombinant *env *of FIV_*Ple *_subtype E, as exclusion of this subtype from the analyses (data not shown) recovered the monophyletic relationship observed with other genome regions (Figure 2A–E).

The predicted *env *protein from both FIV_*Ple *_strains were compared to other published FIV strains with respect to inferred structural elements, with particular focus on regions known to be important for receptor binding. Conserved (white in Figure 4) and variable regions (red in Figure 4) and epitope binding sites (orange in Figure 4) were identified based on their locations in the domestic cat FIV sequences [39]. The V3-V5 regions shared least homology between the two strains. In FIV_*Fca*_, this region has been shown to contain the CXCR4 binding site [40], neutralizing antibody binding sites [41-43] and several epitopes important for cell tropism and cell line adaptation [44-46]. Within the V3-V5 region, several biochemical differences have been noted between domestic and non-domestic cat lentiviruses [39]. FIV_*Ple*_ subtype B demonstrated properties more similar to other non-domestic cat lentiviruses including a negative charge and fewer cysteine residues within this region. Conversely, FIV_*Ple *_subtype E had a positive charge and more cysteines in V3-V5, more similar to the domestic cat lentiviruses. Both lion FIVs had similar numbers of predicted N-glycosylation sites (10 and 11 for B and E, respectively) and these numbers are intermediate to the domestic cat FIVs (8–10) and the other non-domestic FIVs (13–14). A similar trend of lower charge and more cysteine residues in B than E was noted in V3, the region implicated as receptor binding domain for FIV [44,46,47]. In contrast, the more conserved regions flanking V3-V5 were more positively charged in FIV_*Ple *_subtype B than in FIV_*Ple *_subtype E, but contained similar numbers of cysteine residues and putative N-glycosylation sites. Such differences suggest that substantial divergence may occur in secondary and tertiary structures at the receptor-binding region of these two lion lentiviruses.

Recombination in lentiviruses is not uncommon. In the ongoing global HIV pandemic, at least 34 circulating recombinant forms from HIV-1 subtypes have been so far described in patients world-wide [48]. SIV full genome sequence comparisons increasingly depict extant primate lentiviruses with mosaic structures indicative of multiple recombination events over time [49-54]. In FIV_*Fca*_, recombination in the V3-V5 region of *env *was detected between subtypes A and B in feral cats [7], and different recombination frequencies occur between large regions of FIV_*Pco *_subtype B in domestic cat experimentally infected with FIV_*Pco *_B [31]. Whereas the frequency of FIV_*Ple *_recombination is not yet known, our studies show that over 40% of Serengeti lions in Tanzania are multiply infected with FIV_*Ple *_subtypes A, B and C, which circulate freely within this large population [6] and thus offer opportunities for recombination.

The recombination of *env *in FIV infected lions has interesting evolutionary significance because the divergence in this region is extensive between the two subtypes. Therefore, subtype E recombination may represent an ancient event of recombination followed by a long period of divergence, or a more recent recombination with a highly divergent but as yet unsequenced strain either from lions or another African felid species. Although *FIV*_*Ple *_subtype E *env *is more similar to FIV_*Fca *_than to any other known FIV the extent of genetic divergence is still quite substantial, i.e. 64.4% nucleotide relative to FIV_*Fca *_subtype C (Table 2), suggesting that if recombination has occurred recently, it is likely to have been with strain that has not yet been sequenced for the *env *gene. This recombination event may also have functional implications, as *FIV*_*Ple *_subtype E *env *has structural features more similar to pathogenic FIV_*Fca*_. Further investigation into complete genome analyses of *FIV*_*Ple *_subtypes A, C, D and F as well as FIV from other seropositive African felids, will likely provide new insights into the role of recombination in *env *in the wild. Clinical studies will help to clarify the significance of these recombination events.

## Conclusion

Ongoing efforts to sequence full genome FIV from all seropositive exotic cat species will be essential to understanding the evolutionary trajectory of these viruses including the origin and frequency of recombination within FIV. This study demonstrates the necessity of whole-genome analysis to compliment population/gene-based studies, which are of limited utility in uncovering complex events such as recombination that may lead to functional differences in virulence and pathogenicity. The changes observed in the *env *gene as a consequence of recombination in *FIV*_*Ple *_will provide important clues to the natural history of these viruses and their hosts, and may lead to insights into genetic determinants of pathogenicity and virulence differences between domestic cat and lion FIV; findings with important implications for HIV pathogenesis in humans and virus attenuation in wild populations of endangered species.

## Methods

### Cell Culture of FIV_Ple _Subtype E Botswana lion Ple-1027

FIV_*Ple *_subtype E was isolated from PBMCs (whole blood with EDTA) collected from wild lions in the Okavango Delta in Botswana, viably frozen under field conditions [23] and stored in liquid nitrogen. In preparation for cell culture, viably frozen PBMCs from Ple-1027 were thawed at 37°C, washed twice in LBT media (RPMI 1640 (Invitrogen Life Sciences, Carlsbad, Calif.) containing 20% fetal bovine serum (Atlanta Biologicals, Norcross, Ga.), 1% Glutamax I, 1 mM sodium pyruvate, 0.1 mM nonessential amino acids, 5 × 10^-5 ^M β-2-mercaptoethanol, 100 U of penicillin/ml, 100 μg of streptomycin/ml, (all from Invitrogen Life Sciences), and 9 g of glucose (Sigma)/liter), and resuspended at a final concentration of 1–1.5 × 10^6 ^cells/ml in LBT + interleukin-2 at 100 U/ml (Invitrogen Life Sciences).

Domestic cat Mya-1 naïve feeder cells [55] were prepared for co-culture by cultivation in LBT + IL-2, plated in MEM containing 10% fetal bovine serum, 100 U of penicillin, 100 ug of streptomycin/ml, and 1% glutamax, and dispensed at 2 × 10^6 cells/ml in appropriate media in a 24 well plate. Reconstituted lion PBMCs were then added to Mya-1 cells at a volume of 400 ul (4–6 × 10^5 ^cells).

Media was collected biweekly and subjected to microtiter reverse transcriptase assay as follows. Briefly, 15 μl of culture supernatant in triplicate was incubated with 50 μl of 0.05 M Tris (pH 7.8) with 75 mM KCl, 5 mM MgCl_2_, 0.5 mM EGTA, 2 mM dithiothreitol, 5 nM oligo(dT), 0.05% NP-40, poly(A) at 50 μg/ml, and ^32^P at 20 μCi/ml for 90 to 120 minutes at 37°C. Aliquots of 2.5 μl of each reaction mixture were spotted onto a nylon filter (Wallac, Turku, Finland) and allowed to dry. Un-incorporated label was washed away with five 10 to 60 minute washes with 0.03 M sodium citrate, pH 7.0, in 0.3 M sodium chloride (SSC) buffer, and the membrane was then fixed in 100% ethanol. Counts per minute were measured using a Microbeta Counter (Wallac).

Starting on day 34 post co-culture, supernatant from lion PBMC cocultures with Mya-1 cells had reverse transcriptase (RT) values approximately 3 to 10 times naïve supernatant levels, indicating productive lentiviral replication. RT activity was not detected in any other control supernatants through 49 days of culture.

RNA was extracted from 200 μl of supernatant from positive cultures using QIAamp viral RNA mini kit (QIAGEN) and reversed transcribed to cDNA with Superscript II (Invitrogen) according to manufacturer's instructions. PCR was then performed to amplify a diagnostic region of *pol *as previously described [4]. Amplicons were sequenced to confirm the presence of a Botswana strain of FIV (*FIV*_*Ple *_subtype E). One ml aliquots of supernatant were frozen at -70°C. Aliquots were then thawed and used to inoculate 3 × 10^6 ^Mya-1 cells, which were grown 14 days to achieve positive RT values as above. Cells were supplemented with fresh media weekly and grown to 1 × 10^7 ^cells at which point cells were harvested by centrifugation and cell pellets were frozen at -70°C.

### Cell Culture of FIV*_Ple_* Subtype B: Serengeti lion Ple-458

Isolation and culture methods for FIV*_Ple_* Subtype B are similar to the methods described for Subtype E (above) with the following exceptions. FIV*_Ple_* Subtype B was isolated from PBMCs from a wild, sero-positive lion (Ple-458, Serengeti National Park), separated from heparinized whole blood by sucrose gradient centrifugation using Histopaque (Sigma). Cells were mixed with 10% DMSO with 90% fetal calf serum and viably frozen in nitrogen vapor in aliquots of ~10^6 ^cells per ml. Post-freezing, thawed PBMCs (10^6 ^cells) from the wild lion were co-cultivated with an equal number of lion donor cells (Ple-73, captive, National Zoological Park, Wash., D.C.; this lion was sero-positive but had repeatedly tested negative for virus isolation). All PBMCs were mitogen stimulated with concanavalin A (5 ug/ml) for 72 hrs. Co-cultures were propagated in RPMI 1640 with 10% bovine serum and 10% human interleukin-2 (Gibco-BRL). Fresh media was added every 72 hours and new donor cells (10^6 ^cells) were added every 14 days. Replicating virus was confirmed in the supernatant by demonstrating both positive Mg_2_+ – dependent reverse transcriptase (RT) and the presence of typical lentiviral particles seen by electron microscopy [10]. Virus rich supernatants were clarified by slow speed centrifugation and stored in liquid nitrogen freezers.

In order to expand the culture sufficiently to harvest viral supernatant for Western blot assays and to conduct the genetic analysis, 1 ml RT positive supernatant (LLV-2, SV lab) was used to inoculate 3201 cells (5 ml at 2 × 10^6^/ml), FeLV negative lymphosarcoma cells [56]. Cells were maintained in equal parts Leibovitz's L-15 media and RPMI 1640 with 20% fetal calf serum with glutamine (2x) and penicillin/streptomycin (1x). Initially, this culture was difficult to maintain in 3201 cells because it caused rapid cell death thus, in order to keep the culture alive, fresh media and naive 3201 cells had to be added every 3–4 days. After 21 days post infection (dPI), fresh media continued to be added to the culture every 3–4 days, but the addition of naïve 3201 cells was stopped and the % viability was allowed to decline (in the hope that a cell adapted virus could emerge that would enhance our ability to grow up viral stocks for use in Western blot assays). From dPI 28 to 49 the culture viability hovered between 18–24%, but after dPI 52 it was clear that both the viability and cell numbers began to improve (viability from 46 to 86%). By dPI 71 the cell viability was holding at >90% and the culture was growing at 40–50% per day. Infected cells for DNA extraction and genetic analysis of subtype B virus were harvested on dPI 88, centrifuged, and the pellets frozen at -70C.

### DNA extraction, Cloning and Sequencing of FIV_*Ple*_Subtypes B and E

DNA was extracted and purified from frozen cell culture pellets following the manufacturer's protocols established for blood products (Quiagen). Following extraction, DNA quality was checked by gel electrophoresis, and quantified by spectrophotometer (NanoDrop).

*FIV*_*Ple *_proviral DNA was amplified using long PCR to generate overlapping proviral genome regions of approximately 5 kb (Roche's Expand PCR kit). For *FIV*_*Ple *_subtype B, three over-lapping regions were amplified using the followoing primer pairs: FSHltr2F and FIVpol6R (LTR-pol); FIVgag2aF and FIVpol5R (gag-pol); and FIVpol5F and FIVltr4R (pol-LTR) (see Additional file 2). For subtype E, two over-lapping regions were amplified using the two primer pairs FSHltr2F and FIVpol5R (LTR-pol), and FIVgag2aF and FIVltr4R (gag-LTR) (see Additional file 2). PCR reactions used 0.2–2.0 ug DNA with the following thermocycling conditions: 94°C for 2 minutes; ten cycles of 94°C for 10 seconds, 52°C for 30 seconds and 68°C for 4 minutes; 25 cycles of 94°C for 10 seconds, 52°C for 30 seconds and 68°C for 4 minutes and 20 seconds, with each having an extension time 20 seconds longer than the one before it; followed by 68°C for 7 minutes and 4°C hold. Additional "internal" primers were developed to fill in sequence gaps within each subtype (see Additional file 2) using the same PCR conditions listed above. Biometra T1 thermocyclers were used for all PCR reactions and amplicons were visualized on a 1% agarose gel.

The PCR products were cloned using TOPO TA XL cloning kits (Invitrogen). The resultant colonies were grown on LB agar plates with kanamycin and mini-prepped using Qiagen's REAL Prep 96. A restriction digest with EcoRI was performed to confirm successful cloning. The ends of the inserted PCR product were sequenced using the primers provided with the cloning kit for additional verification of *FIV*_*Ple *_cloned products.

The final full-length sequence for each over-lapping long PCR product generated for each *FIV*_*Ple *_subtype was obtained using transposon bombing [GPS-1 kit (New England BioLabs)]. In this method, transposons were randomly inserted into one of the successfully transformed plasmids for each primer combination for each sample. The results were used to transform OneShot Chemically Competent E. coli cells (Invitrogen) and grown on LB agar plates with kanamycin and chloramphenicol and were mini-prepped for DNA extraction using REAL Prep 96 (Quiagen). Restriction digest with EcoRI was performed to confirm successful insertion of the transposon. Using sequencing primers provided in the GPS-1 kit, 48 transposon fragments/PCR reaction were sequenced using an automated sequencer model ABI 3730.

Sequences (average read was approximately 600 bp) randomly generated by transposon bombing of individual clones defined multiple overlapping regions and were assembled into the full-length viral genome using Sequencher version 4.1 (Gene Codes Corporation) and submitted to GenBank [accession number EU117991 (Subtype B) and EU117992 (Subtype E)].

### Genetic and Phylogenetic Analyses of FIV_*Ple*_Subtypes B and E

Gene annotation of *gag*, *pol*, *env*, *orfA*, *vif *and *rev *from open reading frames in lion subtypes B and E used translation into amino acids and comparison with existing full-length FIV strains. The following sequences were used from GenBank: for FIV_*Pco *_Subtype B in pumas strain PLV-1695 accession number DQ192583[27]; for FIV_*Pco *_Subtype A in pumas strain PLV-14 accession number U03982[14]; for FIV_*Oma *_in Pallas cat accession number AY713445[16]; for FIV_*Fca *_subtype C in domestic cat strain C36 accession number AY600517[24]; for FIV_*Fca *_subtype B, strain usil2489, accession number U11820; for FIV_*Fca *_[57]; Subtype A, strain PPR accession number M36968 and strain Petaluma accession number M25381[15,25,58]. Open reading frames were determined and regions of homology between *FIV*_*Ple *_with other FIV strains using pair-wise comparisons implemented by BLAST of two sequences [59]. The boundaries of both the 5'LTR and 3'LTR regions were identified by the conserved polypurine tract (PPT) shared by all FIV [60] and the primer binding site (PBS) which mark the boundary between the 3'LTR and the 5'LTR, respectively.

The genome of lion FIV_*Ple *_was compared with existing full-length FIV by multiple sequence alignments of each viral gene. LTR regions were aligned using Clustal X [61] and verified and edited by eye using Se-Al ver 2.0 [62]. Due to large genetic divergence between FIV from different species, alignment for coding regions of FIV used the program REVTRANS ver 1.4 [29] which takes a multiple sequence file, translates that file into amino acid residues, aligns the amino acids, and uses this alignment as the scaffold for nucleotide alignment. Aligned multiple sequence files were imported into Modeltest ver 3.7 [63] and the optimal model of nucleotide substitution was selected using the AIC criterion (see Additional file 3).

Viral genes were analyzed separately, as well as combined, for genome comparison and phylogenetic reconstruction. Phylogenetic trees based on nucleotide data were obtained using a heuristic search with three different optimality criteria of maximum likelihood (ML), minimum evolution (ME) and maximum parsimony (MP) as implemented in PAUP* ver 4.0b10 [64]. Conditions for the ML analysis included starting trees obtained by stepwise addition, and branch swapping using the tree-bisection-reconnection (TBR) algorithm. Specific conditions for the ME search included starting trees obtained by neighbor – joining, TBR branch-swapping algorithm, and no collapsing of zero-length branches. The MP analyses coded gaps as "missing", with step-wise addition of taxa and TBR branch swapping. Support for nodes within the phylogeny used bootstrap analysis with identical settings established for each method of phylogenetic reconstruction and retention of node bootstrap values greater than 50%. The number of bootstrap iterations consisted of 1000 for ME and MP methods and 100 for ML. Additional analyses were conducted on FIV coding sequences after translation into amino acids. Genetic distances between strains were derived using the Pam-Dayhoff model of amino acid substitution as implemented in MEGA verson 3.1 [65] with gamma-correction (alpha = 2.5) and pairwise deletion of missing data.

## Authors' contributions

JPS conceived the experiments, conducted genetic and phylogenetic analyses, and wrote the paper. CLMcC conducted all PCR and sequencing experiments, analyzed data, and contributed to writing the manuscript. JLT assisted in experimental design and helped write the manuscript. SVW provided cell culture expertise, reagents, and helped write the manuscript. MR collected blood samples from animals in the wild, conducted cell culture of subtype B and helped write the manuscript. KS conducted cell culture of subtype E and helped in writing the manuscript. CW and HW contributed expertise and essential logistic support in obtaining lion blood samples. SJ O'B contributed expertise, reagents, and helped write the manuscript. All authors have read and approved the final version of the manuscript.

## Supplementary Material

Additional file 1 Transcription factors present within FIV_*Ple *_LTR of lion subtypes B and E. These motifs were identified by the setting a threshold similarity score of 85% for screening against the TRANSFAC database at the website . Click here for file

Additional file 2 Primers for lion FIV amplification. The primers span the entire proviral genome of subtype E (Ple1027) and subtype B (Ple458). Shown are the primer sequence, relative position and orientation. Click here for file

Additional file 3 Parameters used in PAUP* analyses for LTR, each viral gene and combined analyses. These parameters were determined using the program Modeltest (see main text) and were implemented for the maximum likelihood and minimum evolution analyses in PAUP. Click here for file
