# Supplementary material for: Genomic organization, sequence divergence, and recombination of feline immunodeficiency virus from lions in the wild
Source: BMC Genomics. 2008 Feb 5;9:66. doi: 10.1186/1471-2164-9-66 (PMC2270836; doi:10.1186/1471-2164-9-66)
Supplement: Additional file 1 — Transcription factors present within FIVPle LTR of lion subtypes B and E. These motifs were identified by the setting a threshold similarity score of 85% for screening against the TRANSFAC database at the website . [file 1471-2164-9-66-S1.doc]

| Description | Name | Position | Score | Database Reference |
| --- | --- | --- | --- | --- |
| Runt-factor AML-1 | AML-1a | 9..14 | 100 | M00271 |
|  |  | 132..127 | 92 |  |
|  |  | 145..140 | 92 |  |
| Ap-1 binding site | AP-1 | 192..184 | 95 | M00199 |
|  |  | 184..192 | 93 |  |
| Activator Protein 4 | AP-4 | 124..115 | 93 | M00175 |
|  |  | 115..124 | 91 |  |
| CdxA | CdxA | 85..79 | 92 | M00101 |
|  |  | 50..56 | 92 |  |
|  |  | 29..35 | 90 |  |
|  |  | 69..63 | 90 |  |
|  |  | 314..308 | 90 |  |
|  |  | 103..97 | 87 |  |
|  |  | 212..206 | 87 |  |
|  |  | 198..192 | 87 |  |
|  |  | 64..70 | 87 |  |
|  |  | 35..29 | 86 |  |
|  |  | 207..214 | 85 |  |
| Nuclear factor of activated T-cells | NF-AT | 108..119 | 92 | M00302 |
|  |  | 366..355 | 88 |  |
|  |  | 291..279 | 85 |  |
| GATA-binding factor 1 | GATA-1 | 178..169 | 91 | M00347 |
| Activator protein 1 | AP-1 | 183..193 | 91 | M00174 |
| Myoblast determining factor | MyoD | 124..115 | 91 | M00184 |
|  |  | 115..124 | 87 |  |
| CdxA | CdxA | 50..56 | 91 | M00100 |
|  |  | 69..63 | 89 |  |
|  |  | 314..308 | 89 |  |
|  |  | 83..89 | 88 |  |
| Sex-determining region Y gene product | SRY | 360..354 | 90 | M00148 |
|  |  | 145..151 | 87 |  |
|  |  | 114..120 | 86 |  |
|  |  | 200..194 | 85 |  |
| GATA binding site | GATA-X | 176..166 | 89 | M00203 |
|  |  | 71..81 | 85 |  |
|  |  | 180..190 | 85 |  |
| Cap signal for transcription initiation | cap | 349..342 | 89 | M00253 |
|  |  | 187..180 | 86 |  |
| Activator protein 4 | AP-4 | 115..124 | 89 | M00176 |
|  |  | 124..115 | 86 |  |
| GATA-binding factor 1 | GATA-1 | 180..167 | 89 | M00127 |
| Ectopic viral integration site 1 encoded factor | Evi-1 | 66..80 | 88 | M00081 |
| Complex of Lmo2 bound to Tal-1, E2A proteins, and GATA-1, half-site 2 | Lmo2 | 177..169 | 88 | M00278 |
|  |  | 179..187 | 87 |  |
| GATA-binding factor 1 | GATA-1 | 177..189 | 88 | M00128 |
|  |  | 179..167 | 87 |  |
|  |  | 239..226 | 85 |  |
| c-Myb | c-Myb | 147..156 | 88 | M00183 |
|  |  | 260..269 | 86 |  |
| Cellular and viral TATA box elements | TATA | 212..226 | 88 | M00252 |
|  |  | 49..35 | 86 |  |
| TCF11/KCR-F1/Nrf1 homodimers | TCF11 | 188..175 | 88 | M00285 |
|  |  | 168..156 | 87 |  |
| c-Ets-1(p54) | c-Ets-1(p54) | 161..149 | 88 | M00074 |
|  |  | 367..355 | 85 |  |
| Upstream stimulating factor | USF | 317..330 | 88 | M00122 |
|  |  | 330..317 | 88 |  |
| Retroviral Poly A downstream element | Poly | 12..20 | 87 | M00211 |
| Octamer binding factor 1 | Oct-1 | 27..40 | 87 | M00162 |
| CRE-binding protein 1/c-Jun heterodimer | CRE-BP1/c-Jun | 165..173 | 87 | M00041 |
| GATA-binding factor 3 | GATA-3 | 62..53 | 87 | M00351 |
| Sterol regulatory element-binding protein 1 | SREBP-1 | 329..319 | 86 | M00220 |
| GATA-binding factor 2 | GATA-2 | 69..78 | 86 | M00076 |
|  |  | 178..187 | 86 |  |
| GATA-binding factor 2 | GATA-2 | 178..169 | 86 | M00348 |
| USF binding site | USF | 327..320 | 86 | M00217 |
| GATA-binding factor 2 | GATA-2 | 178..187 | 86 | M00349 |
| CCAAT/enhancer binding protein | C/EBP | 113..101 | 86 | M00159 |
| Homeo domain factor Nkx-2.5/Csx, tinman homolog | Nkx-2.5 | 208..214 | 86 | M00240 |
| Activator protein 1 | AP-1 | 183..193 | 85 | M00173 |
| Octamer factor 1 | Oct-1 | 113..101 | 85 | M00248 |
| Homeo domain factor Nkx-2.5/Csx, tinman homolog | Nkx-2.5 | 212..205 | 85 | M00241 |
| GATA-binding factor 1 | GATA-1 | 180..167 | 85 | M00216 |
